# Bioinformatics analysis for constructing a cellular senescence-related age-related macular degeneration diagnostic model and identifying relevant disease subtypes to guide treatment

**DOI:** 10.18632/aging.205804

**Published:** 2024-05-10

**Authors:** Shan Luo, Qiang Hu, Bo Jiang, Zhongyu Zhang, Dawei Sun

**Affiliations:** 1 Department of Ophthalmology, The Second Affiliated Hospital of Harbin Medical University, Harbin, Heilongjiang, China

**Keywords:** age-related macular degeneration, cellular senescence, programmed cell death, inflammatory response, molecular subtypes

## Abstract

Age-related macular degeneration (AMD) is a condition causing progressive central vision loss. Growing evidence suggests a link between cellular senescence and AMD. However, the exact mechanism by which cellular senescence leads to AMD remains unclear.

Employing machine learning, we established an AMD diagnostic model. Through unsupervised clustering, two distinct AMD subtypes were identified. GO, KEGG, and GSVA analyses explored the diverse biological functions associated with the two subtypes. By WGCNA, we constructed a coexpression network of differential genes between the subtypes, revealing the regulatory role of hub genes at the level of transcription factors and miRNAs.

We identified 5 genes associated with inflammation for the construction of the AMD diagnostic model. Additionally, we observed that the level of cellular senescence and pathways related to programmed cell death (PCD), such as ferroptosis, necroptosis, and pyroptosis, exhibited higher expression levels in subtype B than A. Immune microenvironments also differed between the subtypes, indicating potentially distinct pathogenic mechanisms and therapeutic targets.

In summary, by leveraging cellular senescence-associated gene expression, we developed an AMD diagnostic model. Furthermore, we identified two subtypes with varying expression patterns of senescence genes, revealing their differential roles in programmed cell death, disease progression, and immune microenvironments within AMD.

## INTRODUCTION

Age-related macular degeneration (AMD) is a disease that affects the macular region of the retina and results in progressive central vision loss [1, 2]. It is estimated that by 2040, 288 million people worldwide will be affected by AMD, with Asia having the highest number of cases (113 million) [3]. Therefore, faster identification of early AMD and provision of better treatments are urgently needed.

The main clinical tests commonly used include fundus color illumination, optical coherence tomography, angiography with optical coherence tomography, and fundus angiography [4]. However, accurate diagnosis with these diagnostic methods is successful only when the pathologic manifestations of AMD are pronounced. In contrast, diagnosis based on gene expression is highly efficient and accurate. Moreover, senescent alterations in multiple retinal and choroidal cell types, encompassing the retinal pigment epithelium, microglia, neurons, and endothelial cells, occurring concurrently with systemic immune aging in both innate and adaptive cells have emerged as significant factors contributing to the initiation and progression of AMD [5]. Therefore, establishing a diagnostic model for AMD based on the transcription of cellular senescence-related genes may predict AMD in advance and benefit the maintenance of patients’ vision. Although there is a consensus on AMD-related clinical typing, most of the existing typing is based on drusen size and the presence or absence of AMD pigmentary abnormalities [6], which has limitations for early prevention and treatment of the disease. Moreover, most of the current treatments for AMD are based on anti-vascular endothelial growth factor therapy (anti-VEGF), which does not allow individualized treatment for each subtype. Furthermore, patients respond differently to anti-VEGF therapy, and not all patients achieve or maintain good vision that is stable over time [7, 8]. Therefore, it is necessary to find new therapeutic targets for AMD. It is also necessary to differentiate patients into distinct subtypes based on molecular characteristics and to identify subtype-specific therapeutic targets for personalized treatment.

The first objective of this investigation was to establish a diagnostic model for AMD based on the transcriptomic profile of cellular senescence-related genes to facilitate earlier disease detection and guide treatment across subtypes. Through machine learning and statistical validation, we established an AMD diagnostic model with robust predictive capabilities. Subsequently, we identified cellular senescence-associated AMD subtypes and extensively explored their molecular and immunological features through transcriptomic analysis. According to the results of unsupervised clustering, we identified two distinct AMD subtypes characterized by differential levels of cellular senescence gene expression. The subtype with elevated cellular senescence levels exhibited a higher degree of immune cell infiltration, increased inflammation levels, and significant involvement in programmed cell death (PCD) and neovascularization processes. Knowledge of the heterogeneity of the transcriptional level of the disease is used to individualize therapeutic strategies for patients. Considering the observed heterogeneity between the subtypes, we identified several promising therapeutic targets. These findings hold the potential to pave the way for individualized treatment approaches for patients afflicted with AMD.

## MATERIALS AND METHODS

### Dataset collection and preprocessing

**Figure 1:**
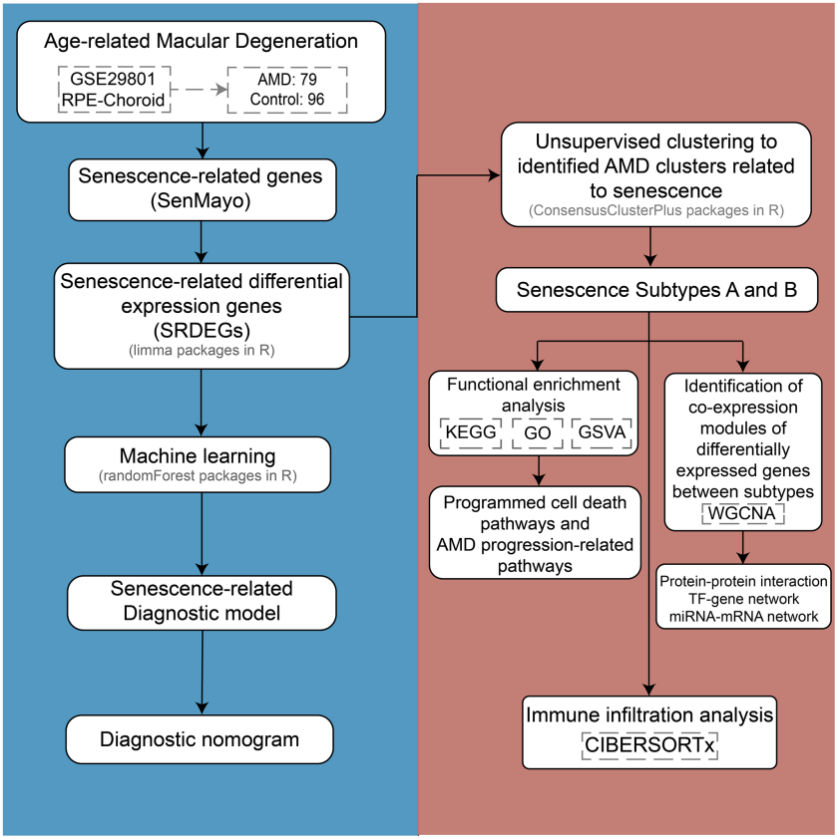
The flowchart of the study design.

The detailed analysis process and analysis tools for this study are shown in Figure 1. In this study, we used the GSE29801 mRNA expression profile dataset from the Gene Expression Omnibus (GEO) database based on the GPL4133 platform. The RPE-choroid tissue samples were retained, and the retinal tissue samples were deleted. A total of 96 samples were collected from the control group, and 79 samples were collected from the AMD group. The normalizeBetweenArrays function in the limma package of R was used for normalization of the dataset and background adjustment.

The senescence gene set ‘Senmayo’ was obtained from the research conducted by Dominik et al. [9]. This set contains 125 senescence-associated genes and is able to identify senescent cells across tissues and species with high fidelity.

### Differential expression analysis of senescence genes between AMD patients and healthy control individuals

We utilized the limma package in R for differential gene expression analysis. The results were visualized through volcano plots, focusing on adjusted *p*-values below 0.05 for statistical significance. Due to a combination of data considerations, we did not limit the logFC values. The association between the obtained differential expression genes and senescence-related genes is presented using a Venn diagram. We refer to genes that are expressed in both gene sets as senescence-related differential expression genes (SRDEGs). Notably, the expression profiles of SRDEGs between the AMD and control groups were represented using a heatmap.

### Random forest algorithm screening for AMD diagnostic markers

The randomForest package in R was used to perform the random forest analysis. The point with the lowest error rate was taken as the optimal parameter mtry (the optimal number of variables in the binary tree in the specified node). The variable importance of the output results (Gini coefficient method) was measured in the process of building the random forest model in terms of reducing the precision and decreasing the mean-square error. We then screened five differential expression genes with the highest importance as risk genes for subsequent analysis.

Logistic analysis of the risk genes was performed using lrm from the rms program package in R, and the results obtained were used to construct the model and plot the nomogram. The receiver operating characteristic curve (ROC) for the training set was plotted, and the area under the ROC curve (AUC) was calculated. External validation was performed using the GSE50195 dataset as the validation set, which consisted of RPE-choroidal tissue samples. The control group had 7 samples, and the AMD group had 9 samples. The ROC curve was plotted for the validation set subjects, and the AUC was calculated.

Subsequently, the accuracy and clinical applicability of the nomogram were tested by the calibration curve, decision curve analysis (DCA), and clinical impact curve (CIC). The GSE50195 dataset serves as a validation set for external validation of the nomogram. The calibration curve, DCA, and CIC of the validation set are plotted.

### Unsupervised clustering to establish cellular senescence-associated AMD subtypes

Based on the expression of 16 SRDEGs, we performed unsupervised clustering with 1000 replications using the ConsensusClusterPlus package in R. The most suitable cluster count was determined by identifying the K value associated with the lowest proportion of ambiguously clustered pairs [10], as outlined in the literature and the reference manual of the R package.

### Functional enrichment analysis for differential expression genes

Genes showing significant differences between different AMD subtypes associated with cellular senescence were identified using the limma package in R [11]. Specifically, genes with |Log2FC (fold-change) | > 1 and adjusted *p*-value < 0.05 were considered significantly different. Among these genes, some genes were simultaneously expressed in the senescence gene set, which we identified as differential expression genes (DEGs).

GO and KEGG enrichment analyses of DEGs in the two subtypes were performed using Metascape. In addition, gene set variation analysis (GSVA) was used to assess the biological functions and progression variants of these cellular senescence-related genes [12]. The Reactome gene set (c2.cp.reactome.v2023.1.Hs.symbols.gmt) was downloaded from MSigDB for running GSVA. Displaying only the first 20 pathways with adjusted *p*-values < 0.05. GSVA was used to evaluate different AMD subtypes based on biological functions associated with cellular senescence.

### Differential expression analysis of angiogenesis and PCD pathways among different subtypes of AMD

We obtained gene sets for angiogenesis, pyroptosis, necroptosis, and mitophagy by downloading them from MSigDB. The ferroptosis gene set was obtained from the FerrDb database [13]. The cuproptosis gene set was extracted from a previous study [14]. Gene differential expression analysis was performed using the limma package in R and visualized using heatmaps and box plots. Adjusted *p*-values less than 0.05 were considered statistically significant.

### Immune infiltration analysis

Gene expression data from all samples of both subtypes A and B were deconvoluted using the CIBERSORT algorithm [15] to calculate the composition of the 22 immune cell infiltrates. Differences in immune cell proportions between the two subtypes were compared using the Wilcoxon rank sum test.

### Identification of cellular senescence-associated hub genes

Weighted gene coexpression network analysis (WGCNA) was performed with the WGCNA package in R to obtain the coexpression gene modules most highly correlated with the differentiation of the two subtypes [16]. Coexpression networks were constructed using genes from RPE-choroid tissue samples in the normalized GSE29801 dataset. First, the cut height was set to 140, and we detected and excluded outliers by sample clustering. The Pearson coefficients between individual genes were calculated to transform them into similarity matrices, and the network topology analysis was automatically performed by the pick soft threshold function of the WGCNA package to select the soft threshold β, which can emphasize the strong and weak correlations between genes. After determining β, the similarity matrix was transformed into an adjacency matrix, and then the adjacency matrix was transformed into a topological overlap matrix (TOM). Subsequently, the minimum number of genes in each module was set to 50, and the shear height was 0.2. The coexpressed gene modules obtained by the dynamic shear tree were merged with similar modules. Finally, the Pearson correlation coefficients between gene modules and subtypes A and B were calculated, and the modules that were highly correlated with the subtypes were selected as candidate modules. Intramodule genes were identified as module genes related to cellular senescence. The relationship between genes and modules was measured by calculating the KME value (module eigengene-based connectivity), and |kME| > 0.8 was selected to screen out important genes.

The STRING database (https://string-db.org/) was used to build protein-protein interaction (PPI) networks for important genes, and confidence scores higher than 0.7 were considered significant correlations. The results were visualized and analyzed in Cytoscape software. The cytoHubba plugin was used to screen the hub genes among the upregulated and downregulated genes of important genes. The MCC algorithm was considered the most effective method to find the hub nodes in the coexpression network. The MCC of each node was calculated by the plugin cytoHubba in Cytoscape. In subtype B, the top ten nodes were regarded as hub genes. In subtype A, all the hub nodes were regarded as hub genes.

### Construction of transcriptional regulatory networks of hub genes related to cellular senescence

NetworkAnalyst (https://www.networkanalyst.ca/) is a comprehensive network visualization and analysis platform for gene expression analysis. The miRNA and transcription factor (TF) prediction for hub genes was performed using the miRTarBase v8.0 database and the JASPAR database, respectively. The TF-hub genes and hub gene-miRNA interaction networks were further visualized in Cytoscape 3.9.1 software.

### Data availability statement

The datasets and source codes used or analyzed during the current study are available from the corresponding author upon reasonable request.

## RESULTS

### Differential expression analysis

**Figure 2:**
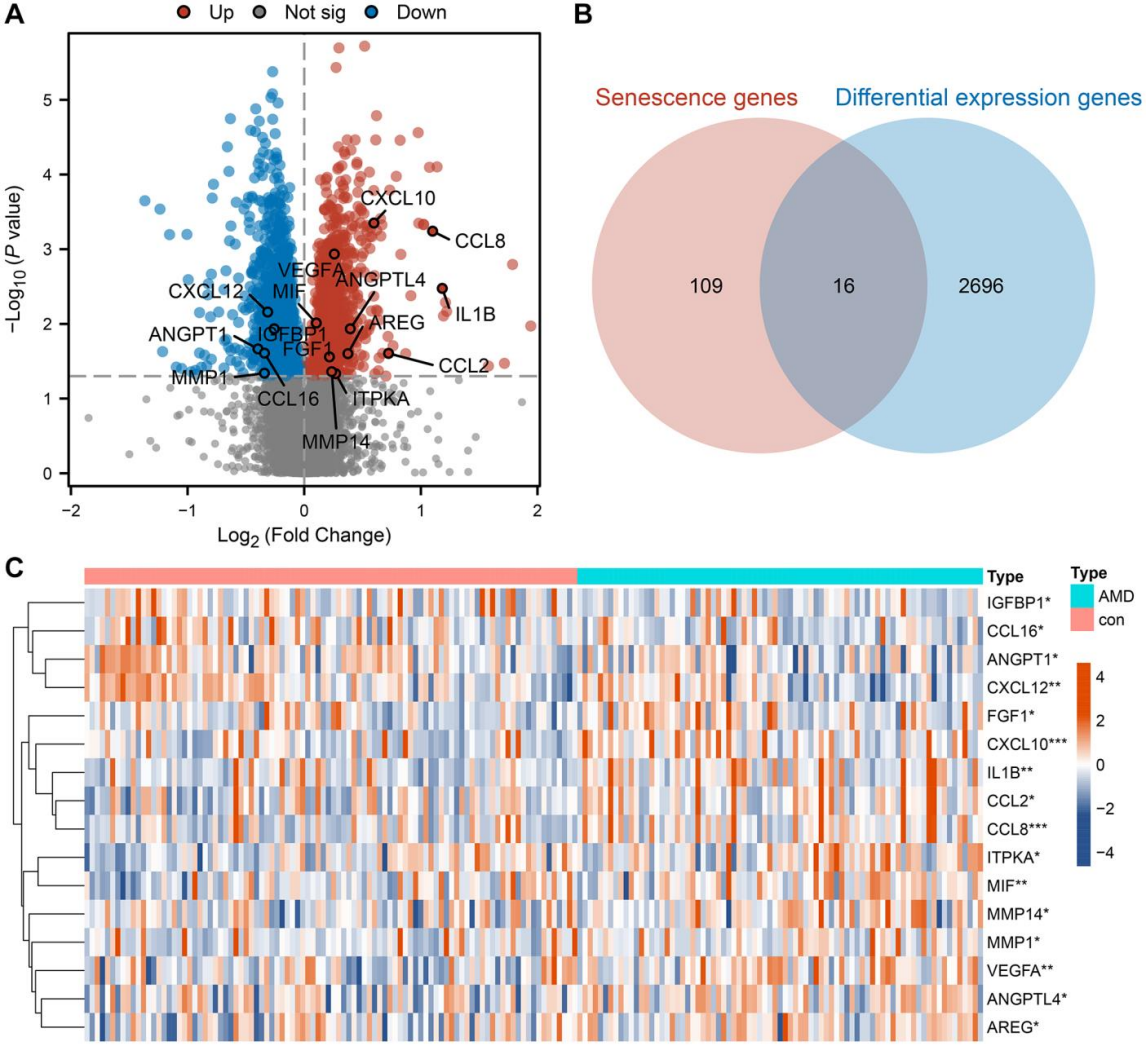
**Identification of differential expression cellular senescence genes in AMD.** (**A**) Differential expression volcano plot between the AMD and control groups. The red dot indicates up-regulated genes in the AMD group, the blue dot indicates down-regulated genes and the grey dot indicates the difference is not significant. (**B**) The intersection of senescence genes and differential expression genes between AMD and the control group. (**C**) Expression heatmap of SRDEGs in AMD and control sample. Red represents upregulation and blue represents downregulation. (^*^P < 0.05; ^**^P < 0.01; ^***^P < 0.001).

Differential expression analysis was performed based on the microarray dataset GSE29801 to screen for senescence-related differential expression genes. We deleted duplicate sequences and samples from retinal tissues in this dataset and retained samples from RPE-choroid tissues, yielding a total of 96 samples in the control group and 79 samples in the AMD group. Next, the limma package in R was used to identify differential expression genes between AMD samples and normal control samples of this microarray dataset. The result is shown in the volcano plot (Figure 2A). Based on the adjusted significance threshold of *P* < 0.05, we identified 2712 significantly differential expression genes associated with AMD disease. The relationship between these differential expression genes and senescence genes is shown in the Venn diagram (Figure 2B), which shows that there are 16 SRDEGs. The expression of SRDEGs between the AMD and control groups is shown in the heatmap (Figure 2C).

### Random forest algorithm screening for AMD diagnostic markers

**Figure 3:**
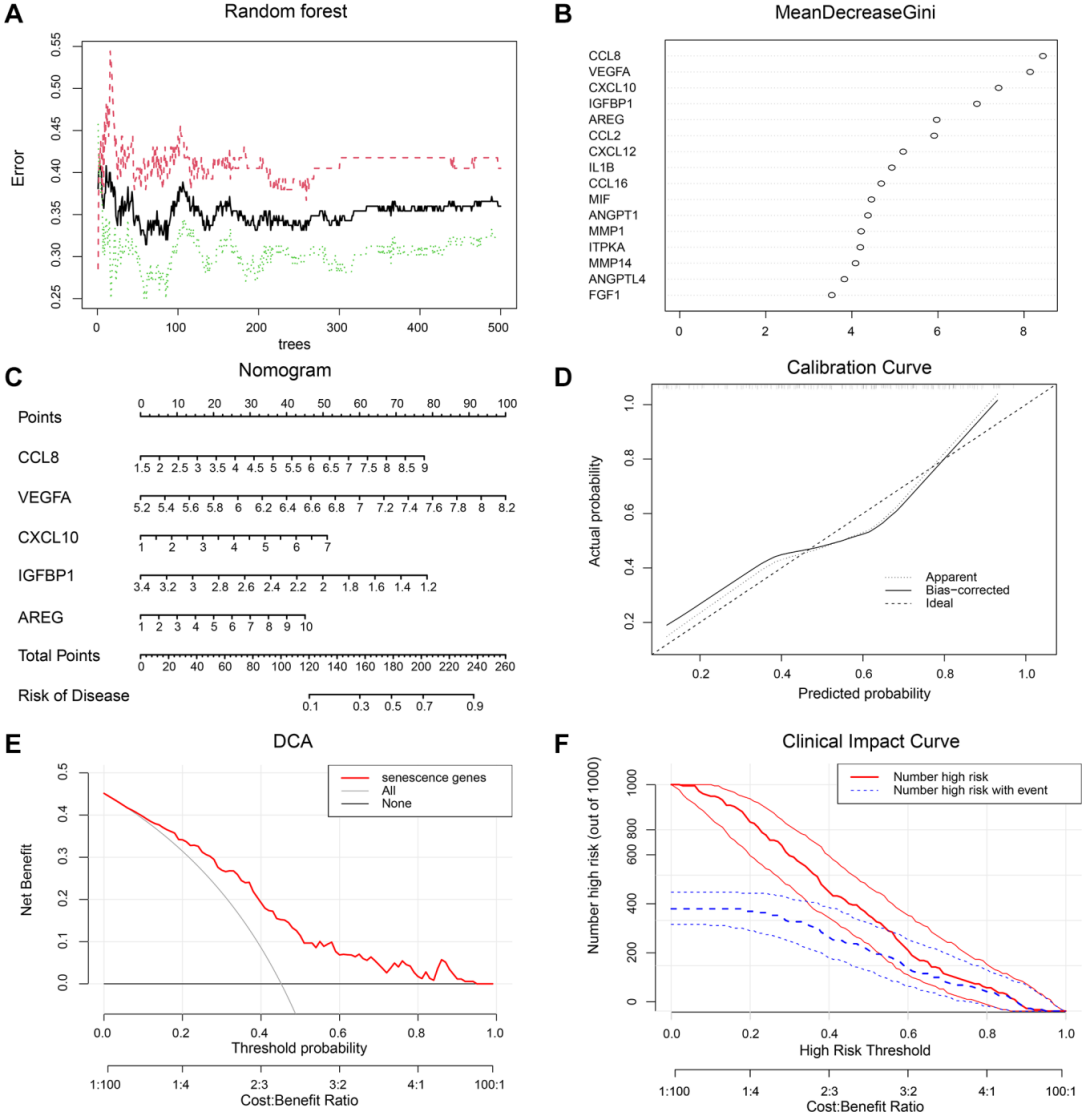
**A diagnostic model was constructed with SRDEGs.** (**A**) Random forest out-of-bag error curve. (**B**) Gini importance scores of candidate genes. (**C**) Nomogram of key genes to predict the risk for AMD. The expression of five risk genes, CCL8, VEGFA, CXCL10, IGFBP1, and AREG, was used as the axis. A straight line was drawn on the ‘Points’ axis to determine the scores related to these genes. The resulting scores were summed and the total score was placed on the ‘Total Points’ axis. A straight line was drawn down the ‘Risk of Disease’ axis to obtain the risk of developing AMD. (**D**) Calibration curve of the diagnostic model. (**E**) Decision curve analysis of the diagnostic model. (**F**) Clinical impact curve of the diagnostic model.

Based on SRDEGs, a random forest model was built. Using a random seed, the number of candidate feature subsets (mtry) was 4, and the number of fixed decision trees (ntree) was 1–500. The variation in the average out-of-bag estimation error rate with the ntree was observed (Figure 3A). The average out-of-bag estimation error rate was lowest when the ntree was 60. Therefore, the number of decision trees at a ntree of 60 was selected for this study to obtain the optimal model. The importance ranking of the explanatory variables was obtained based on the average decrease in the Gini coefficient for each risk factor in the model (Figure 3B). The top 5 genes used to predict AMD in random forest filtered by variable importance measures were as follows: CCL8, VEGFA, CXCL10, IGFBP1, and AREG. Plot the ROC of the training set subjects (Supplementary Figure 1A) and calculate the AUC as 1. Plot the ROC of the validation set subjects (Supplementary Figure 1B) and calculate the AUC as 0.770. The model has a better ability to detect and diagnose AMD.

Logistic analysis of the hub genes was performed using lrm from the rms package in R. The results obtained were used to construct the model and plot the nomogram. Column line plots were used for predicting the probability of AMD. Based on the final regression analysis, graphical column line plots constructed from the final model included the five risk genes for predicting the probability of AMD. The risk factors CCL8, VEGFA, CXCL10, IGFBP1, and AREG were used to calculate the total score. The value of each variable is given a certain score on a scale of quartiles from 0 to 100. The total score can be calculated by summing the scores for each factor. Then, by projecting the total score onto the total score scale axis at the bottom, we can predict the probability of AMD (Figure 3C).

The calibration curve was then used to evaluate the predictive power of the nomogram model. The calibration curves show that the error between the actual risk of AMD and the predicted risk is very small, indicating that the nomogram model predicts AMD with a high degree of accuracy (Figure 3D). Decision curve analysis (DCA) showed that the nomogram curve was higher than the gray line, suggesting that the clinical benefit of the nomogram model was higher at the high-risk threshold of 0–1 (Figure 3E). To evaluate the clinical effect of the nomogram model more intuitively, a clinical impact curve was drawn based on the DCA curve. Under the high-risk threshold of 0.4–1, the “number of high risks” curve was very close to the “number of high risks with events” curve, which indicated that the nomogram model had extraordinary predictive ability (Figure 3F). The calibration curve (Supplementary Figure 1C), DCA (Supplementary Figure 1D), and CIC (Supplementary Figure 1E) plotted in the external validation of the nomogram show that the nomogram has good recognition ability.

These results also suggest to some extent that these five genes may play a key role in the development of AMD.

### Identification of distinct senescent clusters in AMD

**Figure 4:**
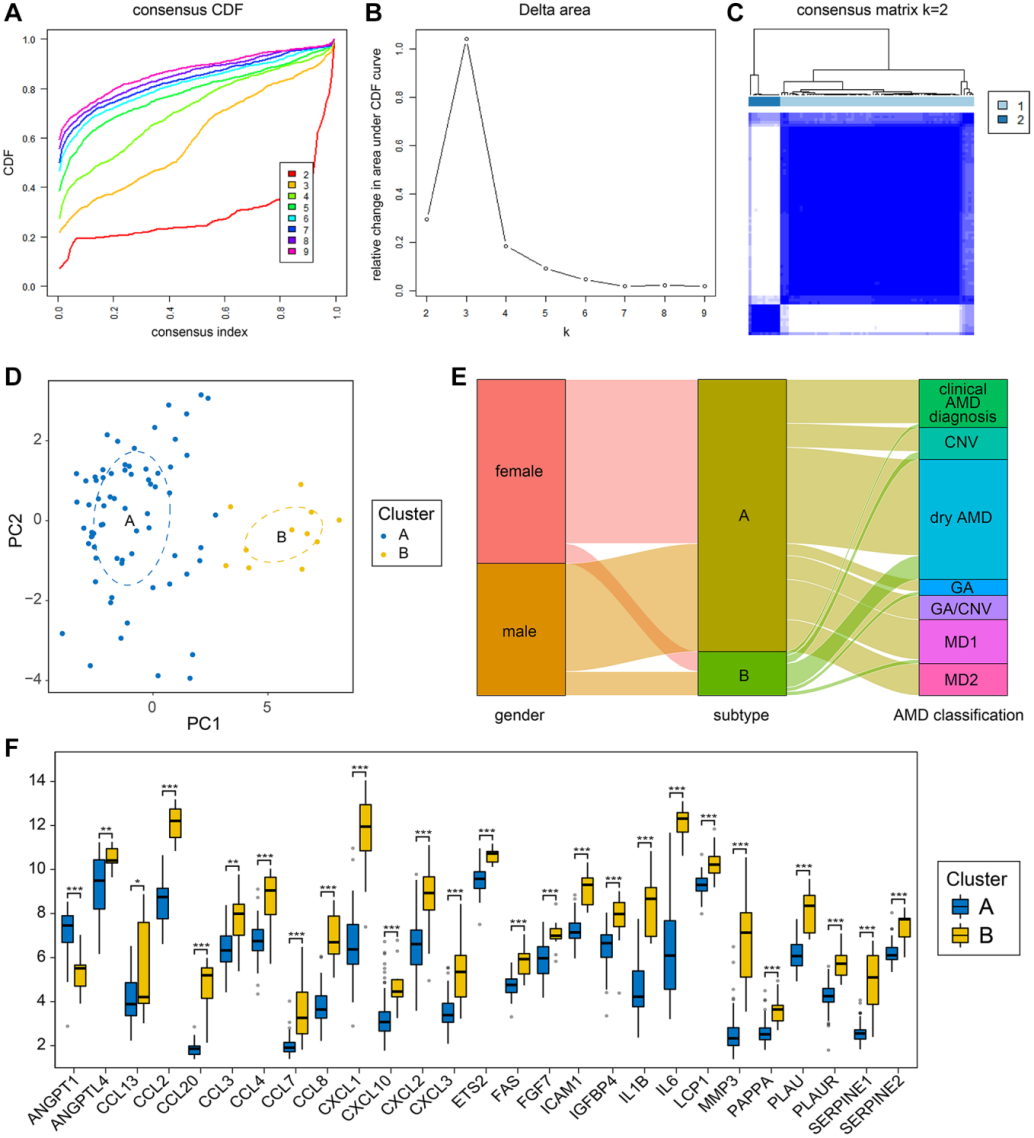
**Cellular senescence-related subtypes established by unsupervised clustering.** (**A**) The CDF curves illustrate the cumulative distribution functions for varying values of K, with the clustering effectiveness being most reliable when K = 2. (**B**) Delta area plots for different values of K during the clustering process. (**C**) Clustering heatmap for unsupervised clustering when k = 2. (**D**) PCA illustrates the classification outcomes of the two subtypes. (**E**) Sankey diagram comparing clinical grouping styles and subtype grouping styles of AMD samples. (**F**) Senescence-related differential expression genes grouping comparison between the two subtypes. (^*^P < 0.05; ^**^P < 0.01; ^***^P < 0.001).

Unsupervised hierarchical cluster analysis of the AMD disease group samples based on 16 SRDEGs was used to examine multiple differential genes for AMD. In the CDF (cumulative distribution function), the curve (Figure 4A) was smooth and stable when K = 2. The delta area (Figure 4B) also shows that it can be reliably divided into two clusters. A total of two distinct AMD subtypes were identified (Figure 4C), of which 68 samples belonged to subtype A, and 11 samples belonged to subtype B. Principal component analysis (PCA) (Figure 4D) indicated that the division of the samples into two groups was successful. The two AMD subtypes identified were inconsistent with the current clinical classification system which focuses mainly on patient symptoms (Figure 4E).

In addition, difference analysis was performed between subtypes A and B, and 410 DEGs were obtained. A total of 296 upregulated genes and 114 downregulated genes were found in subtype B relative to subtype A. We took the intersection set between the DEGs and the senescence-related genes and obtained 27 senescence-related differential expression genes. We used the Mann-Whitney *U*-test (Wilcoxon rank-sum test) to compare the expression levels of these 27 genes between subtypes A and B, and histograms were plotted for the groups (Figure 4F). As shown in the figure, the expression levels of 26 genes, all except ANGPT1, were higher in the B subtype, indicating that the degree of cellular senescence in the B subtype samples was higher.

### GO/Kyoto encyclopedia of genes and genomes (KEGG) enrichment analysis

Based on the DEGs expression results, Metascape was utilized to predict and explore cellular senescence-related biological functions and pathway enrichment between the A and B subtypes using GO and KEGG methods.

**Figure 5:**
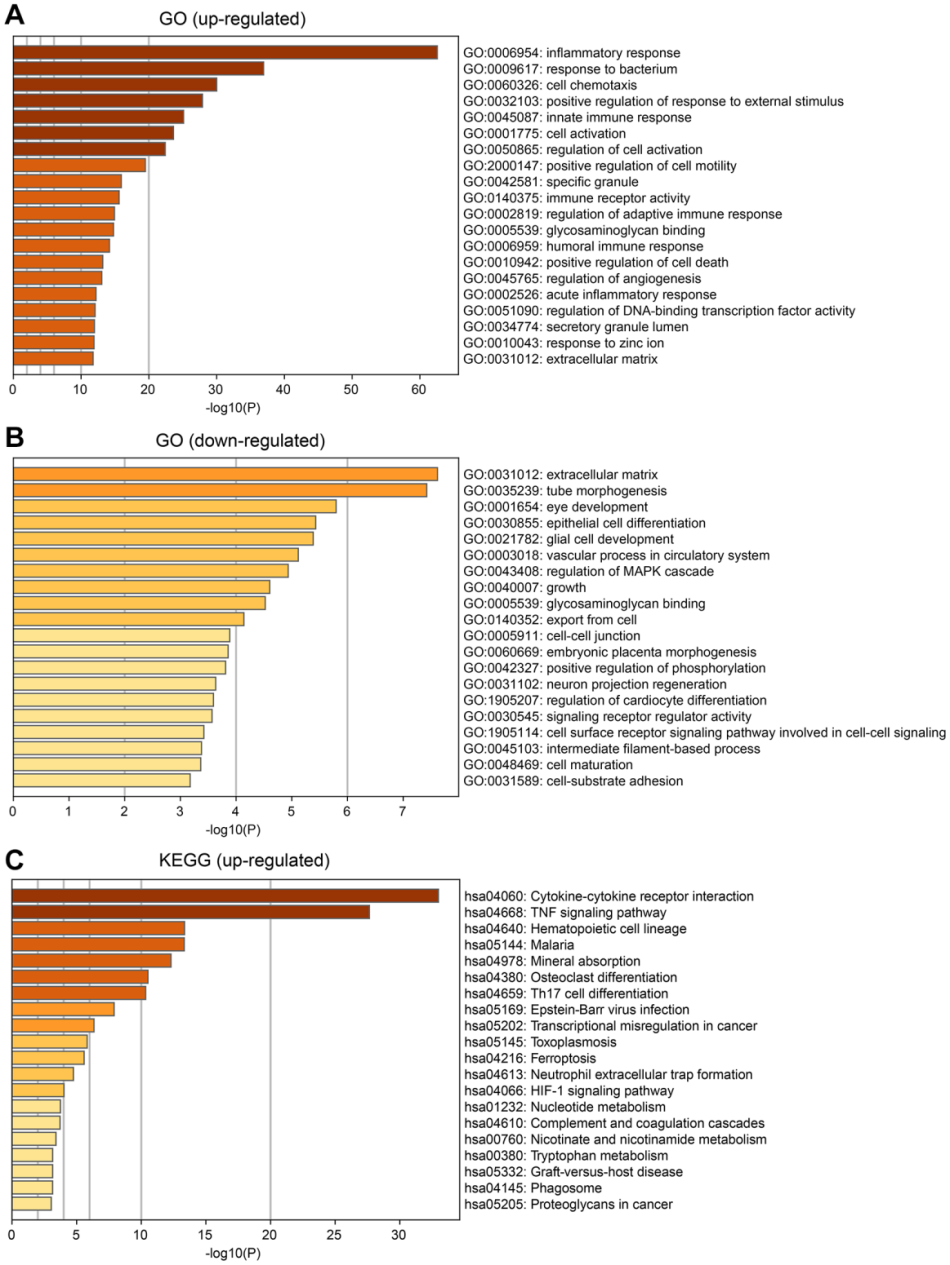
**Enrichment analysis of differential expression genes between the two subtypes.** (**A**) Gene Ontology (GO) enrichment analysis for up-regulated DEGs. (**B**) GO enrichment analysis for down-regulated DEGs. (**C**) KEGG analysis for up-regulated DEGs.

The upregulated and downregulated genes in the GO enrichment analysis are shown in the figures (Figure 5A). The upregulated genes of DEGs in the B subtype were mostly enriched in the inflammatory response, bacterial response, cell chemotaxis, and immune response. The downregulated genes were mostly enriched in the extracellular matrix, eye development, epithelial cell differentiation, and cell maturation (Figure 5B). KEGG pathway enrichment analysis of the upregulated genes is shown in the figures (Figure 5C). The upregulated pathways include cytokine-cytokine receptor interactions, the TNF signaling pathway, and ferroptosis.

### Analysis of GSVA enrichment among different subtypes

**Figure 6:**
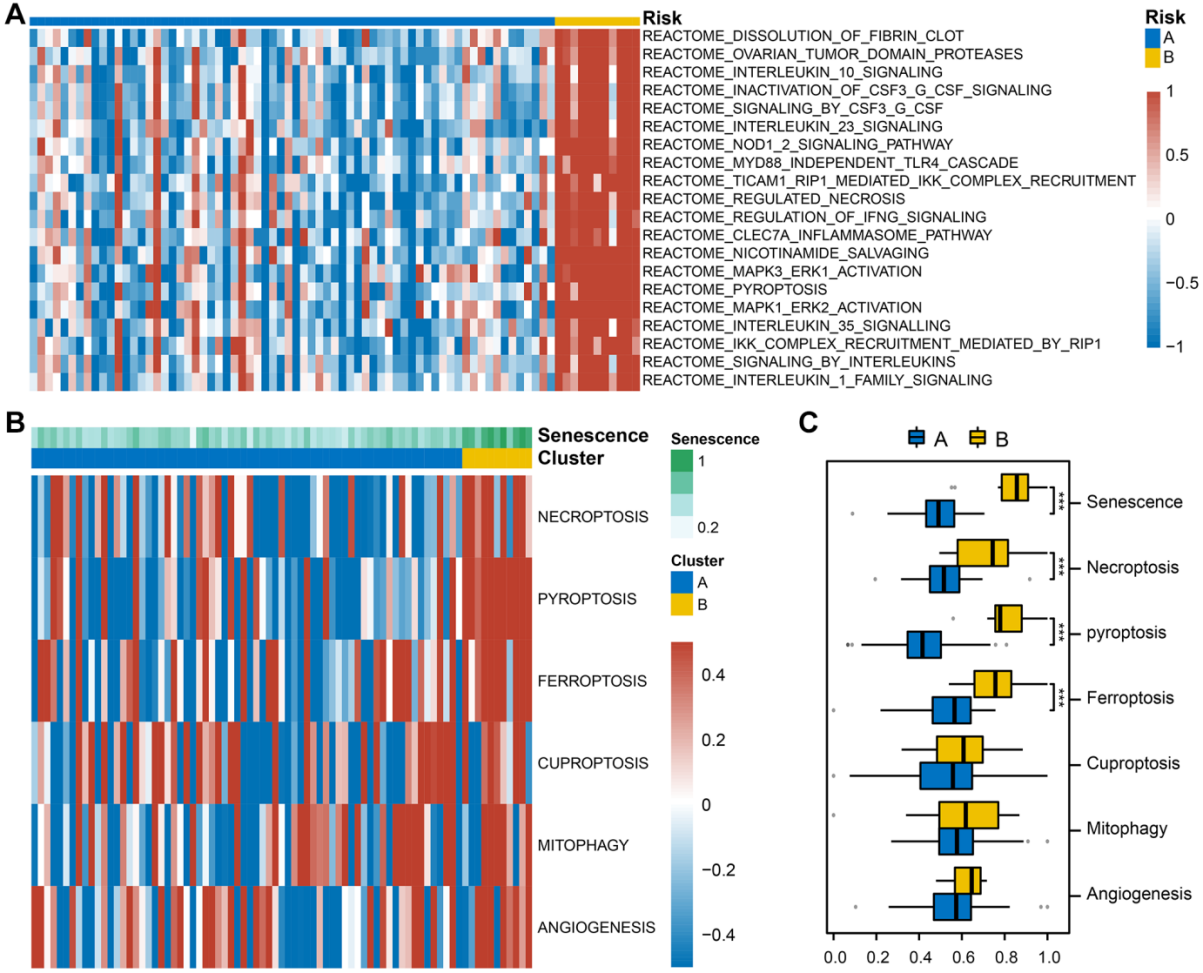
**Gene set variation analysis (GSVA) between the two subtypes.** (**A**) The heatmap illustrates the enrichment patterns of two subtypes. (**B**) Based on GSVA scores, group comparisons of senescence, PCD, and angiogenesis in the two subtypes. (**C**) Box plot with Wilcoxon rank sum test was performed to assess significant statistical differences between subtypes (Abbreviation: ns: not significant; ^*^P < 0.05; ^**^P < 0.01; ^***^P < 0.001). Blue indicates subtype A, and yellow indicates subtype B.

To investigate the biological differences between the two subtypes, we used GSVA enrichment analysis to explore the Reactome pathways associated with each subtype. Compared with type A, type B was mainly enriched for signaling pathways such as inflammation and programmed cell death (PCD) (Figure 6A). In addition, pathways such as dissolution of fibrin clots and ovarian tumor domain proteases were found to be enriched in type B, suggesting that cellular senescence may also be involved in the pathogenesis of tumor-related diseases.

### Differential expression of programmed cell death and neovascularization among different subtypes

Because the two cellular senescence-associated AMD subtypes are strongly associated with programmed cell death (PCD), we analyzed the differences in the expression levels of genes involved in necroptosis, pyroptosis, ferroptosis, cuproptosis, and mitophagy to identify the PCD-related expression patterns of the different subtypes. In addition, it is common for AMD to develop into wet AMD, i.e., neovascular AMD, at a later stage of clinical development. Therefore, we also performed differential expression analysis of genes causing the appearance of angiogenesis among different subtypes. Related heatmaps and box plots are shown in the Supplementary Figures 2 and 3. The GSVA score was used to assess the expression of genes related to the above multiple PCD and angiogenesis pathways and other pathways in the two subtypes A and B (Figure 6B). As shown in Figure 6C, the differences in the expression levels of genes related to senescence, ferroptosis, necroptosis, and pyroptosis in the two subtypes were significant, and the score of the B subtype was higher than that of the A subtype.

### Immune infiltration analysis

**Figure 7:**
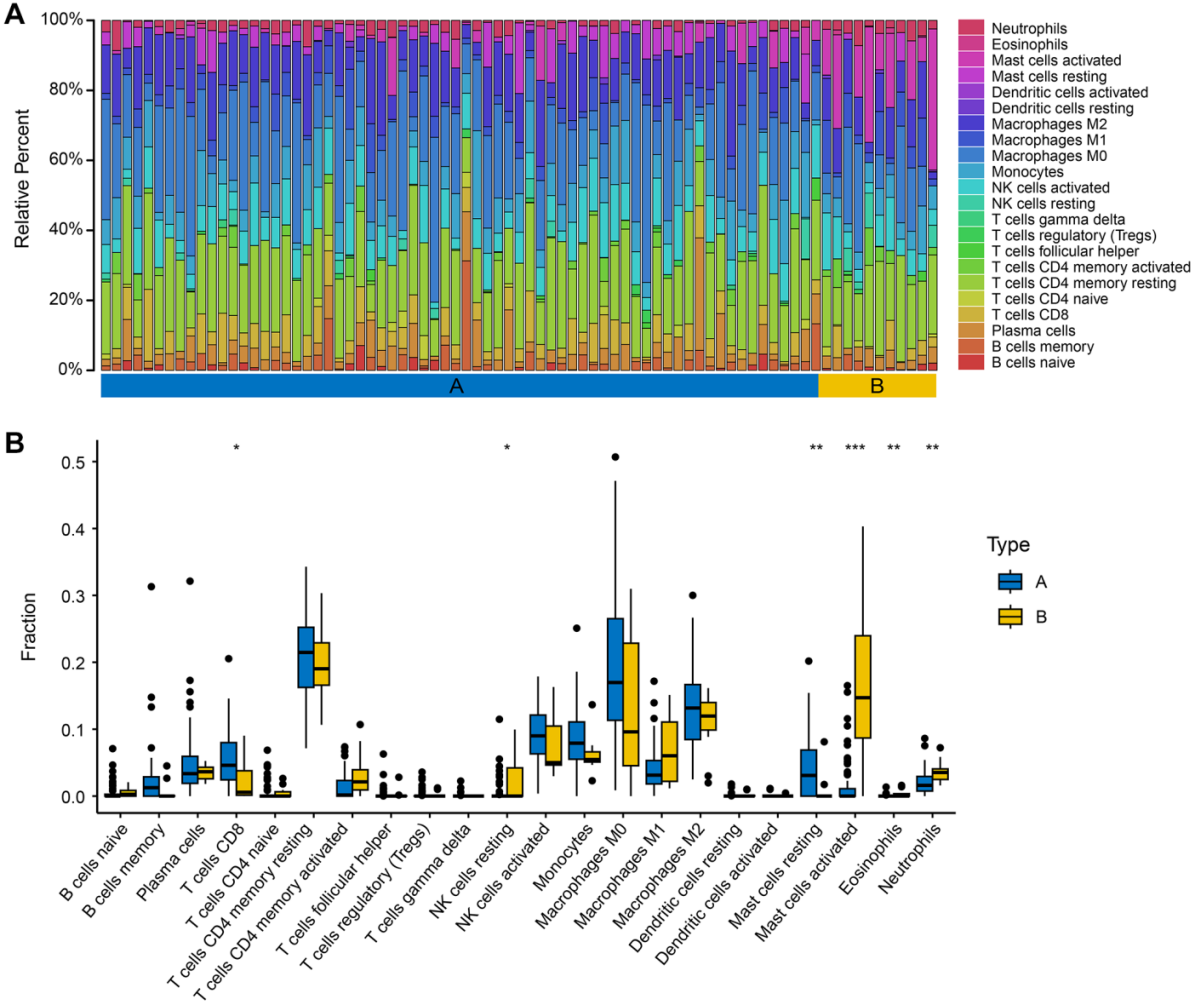
**Immune infiltration characteristics of the two subtypes.** (**A**) The relative percent of 22 immune cells of the two subgroups. (**B**) The different fractions of immune cells in two AMD subgroups. The scattered dots represent the immune cell fraction. The thick lines represent the median value. The bottom and top of the boxes are the 25 and 75 percentiles, respectively. “^*^” is used to represent significant statistical differences between the two subgroups (^*^P < 0.05; ^**^P < 0.01; ^***^P < 0.001).

We utilized CIBERSORTx to analyze samples from both subgroups and derive the proportions of immune cell infiltration (Figure 7A). CD8 T cells, resting memory CD4 T cells, activated NK cells, monocytes, M0 macrophages, M1 macrophages, M2 macrophages, and activated mast cells were more abundant in both subtypes. There were significant differences in the abundances of CD8 T cells, resting NK cells, resting mast cells, activated mast cells, eosinophils, and neutrophils in the two isoforms (Figure 7B). Among them, the abundances of CD8 T cells and resting mast cells were higher in subtype A, and the abundances of activated mast cells and neutrophils were higher in subtype B. These findings may indicate that the abundance of immune cells of lymphoid lineage is higher in subtype A, and the abundance of immune cells of myeloid lineage is higher in subtype B. This may indicate that subtypes A and B exist in different immune environments.

### Cellular senescence-related coexpression hub genes

**Figure 8:**
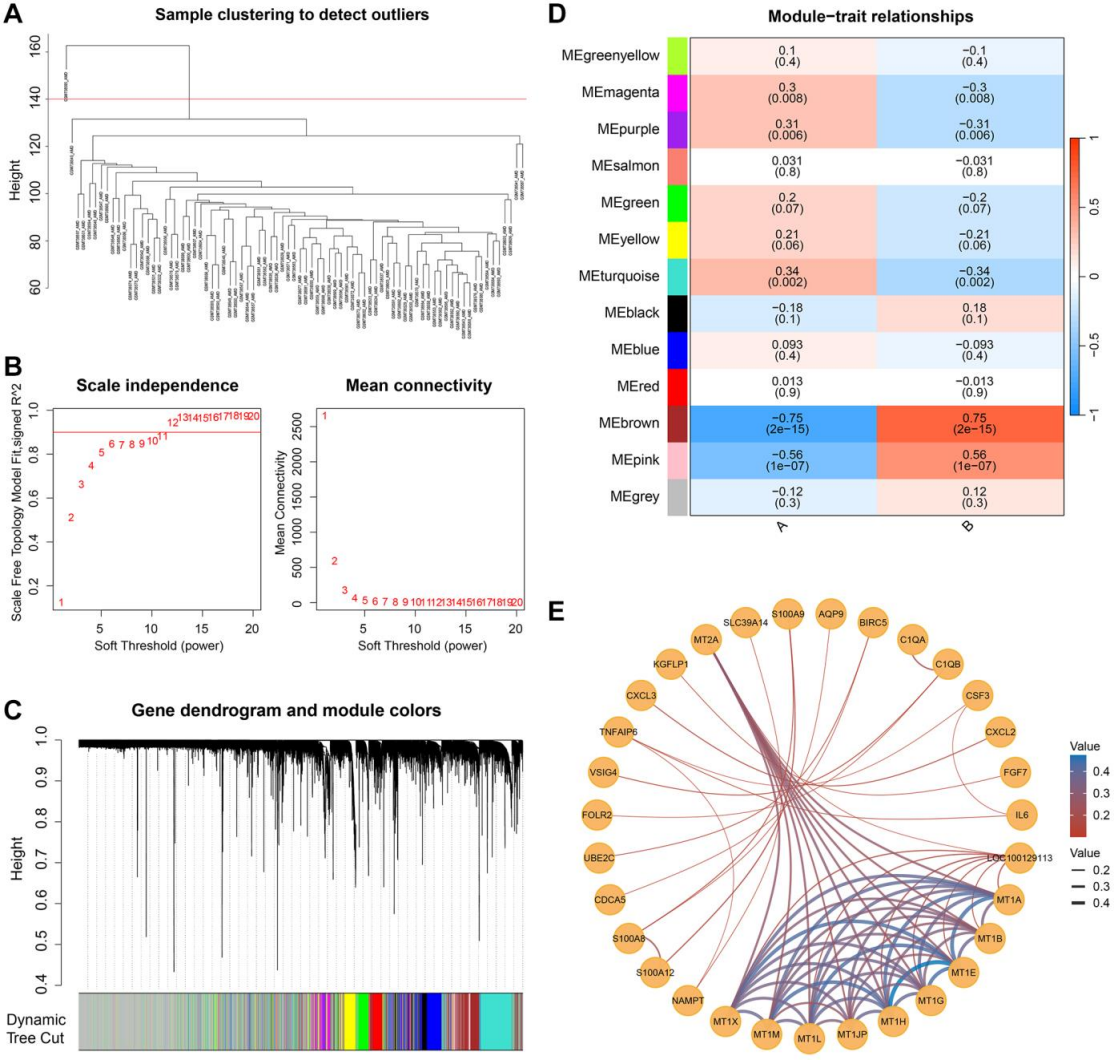
**Weighted gene co-expression network analysis (WGCNA) identified cell senescence-related hub genes.** (**A**) One outlier was cut by setting the cut height at 140. (**B**) Determine the optimal soft threshold of WGCNA by scale independence and average connectivity. (**C**) Dendrogram and module colors of genes in the WGCNA process. (**D**) Twelve non-gray modules and their correlation with subtypes. (**E**) The network of the genes in the brown module.

Coexpression networks were constructed using genes from RPE-choroid tissue samples normalized in the GSE29801 dataset to obtain hub genes between different cellular senescence-related isoforms. Thirteen coexpression modules were identified based on the dynamic shear tree after excluding one outlier sample (Figure 8A) and setting the power value to an optimal soft threshold of 10 (Figure 8B) (Figure 8C). The Brown module (r = 0.75, *P* = 2e-15) had the largest value of differential correlation in subtype B, and the turquoise module (r = 0.34, *P* = 0.002) has the largest value in subtype A (Figure 8D).

**Figure 9:**
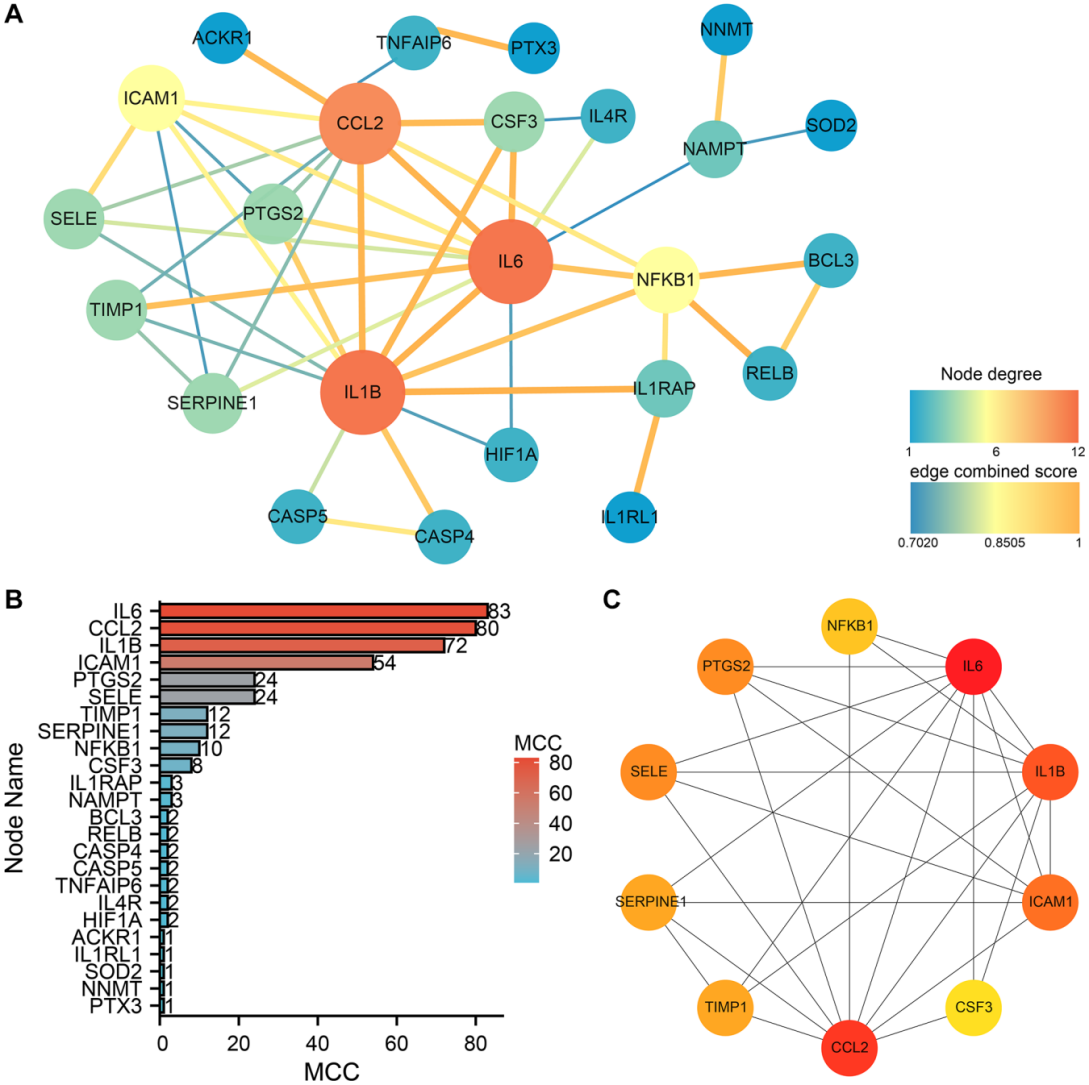
**PPI network construction and analysis of subtype B.** (**A**) The PPI network of the 24 hub genes. (**B**) MCC ranking based on the degree of nodes. (**C**) The top ten genes ranked by MCC, exhibit an increase in MCC scores as indicated by the deepening color.

The relationship between genes and modules was measured by calculating the KME value (module eigengene-based connectivity). |kME| > 0.8 was chosen to screen out 72 important genes in subtype B, and construct gene coexpression networks (Figure 8E). We constructed a protein-protein interaction (PPI) network for hub genes using the STRING database, with a confidence score threshold set at 0.7 for significant associations. In subtype B, the resulting PPI network, consisting of 24 nodes and 46 edges, was visualized using Cytoscape software, with node sizes proportional to their degree (Figure 9A). Hub genes were selected from the PPI network by the MCC algorithm utilizing the CytoHubba plugin (Figure 9B). Based on the MCC score, IL6, CCL2, IL1B, ICAM1, PTGS2, SELE, TIMP1, SERPINE1, NFKB1, and CSF3, which were ranked in the top ten genes of the scores (Figure 9C), were selected as hub genes.

**Figure 10:**
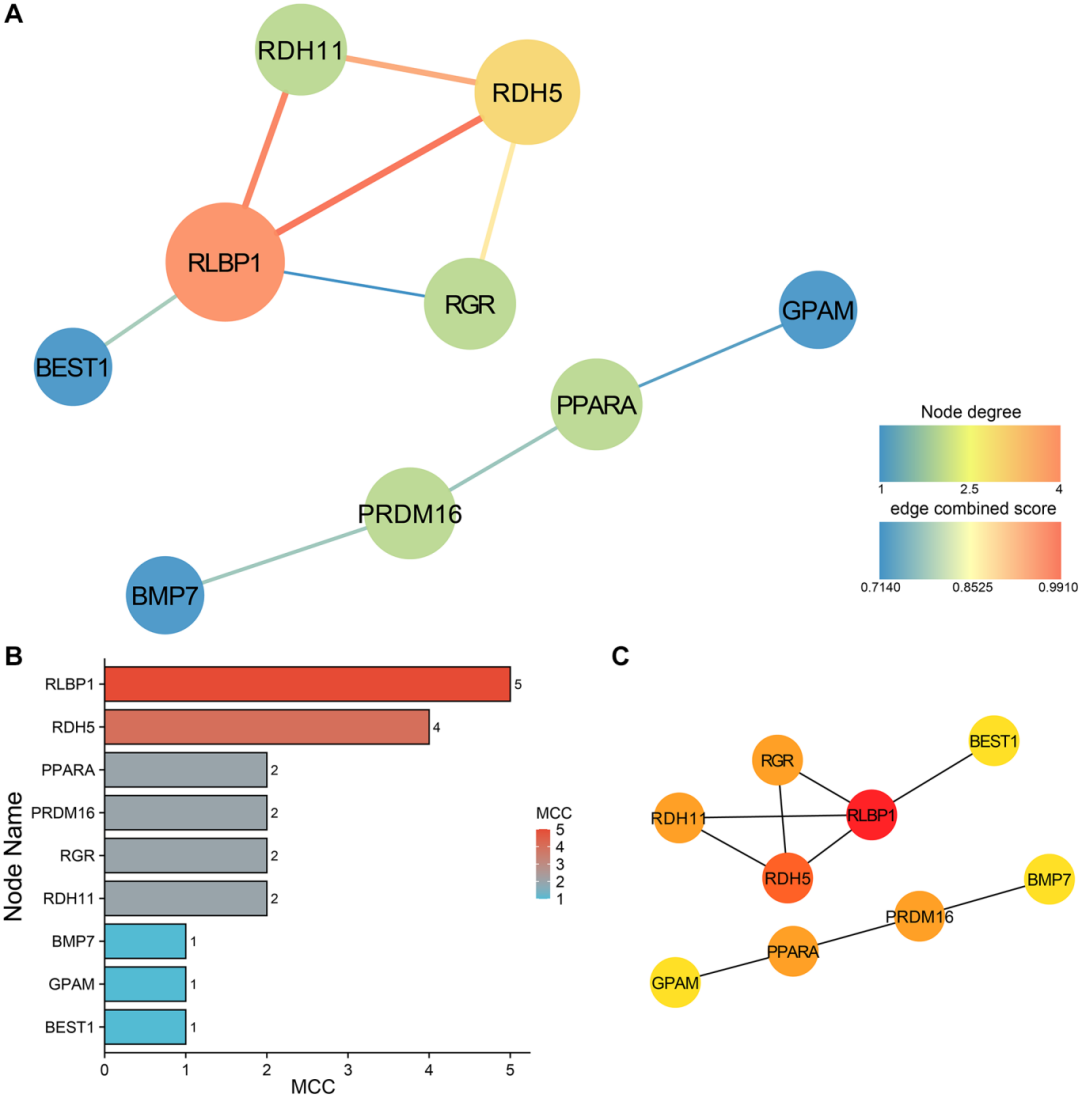
**PPI network construction and analysis of subtype A.** (**A**) The PPI network of the 9 hub genes. (**B**) MCC ranking based on the degree of nodes. (**C**) The genes ranked by MCC, exhibit an increase in MCC scores as indicated by the deepening color.

We also chose |kME| > 0.8 to screen out 123 important genes in subtype A. In subtype A, the resulting PPI network, consisting of two small networks, was visualized using Cytoscape software, with node sizes proportional to their degree (Figure 10A). Hub genes were selected from the PPI network by the MCC algorithm utilizing the CytoHubba plugin (Figure 10B). All the nodes were regarded as hub genes, including RLBP1, RDH5, RDH11, RGR, PRDM16, PPARA, BEST1, GPAM and BMP7(Figure 10C).

### Prediction of miRNAs and transcription factors associated with senescence-related hub genes among subtypes

**Figure 11:**
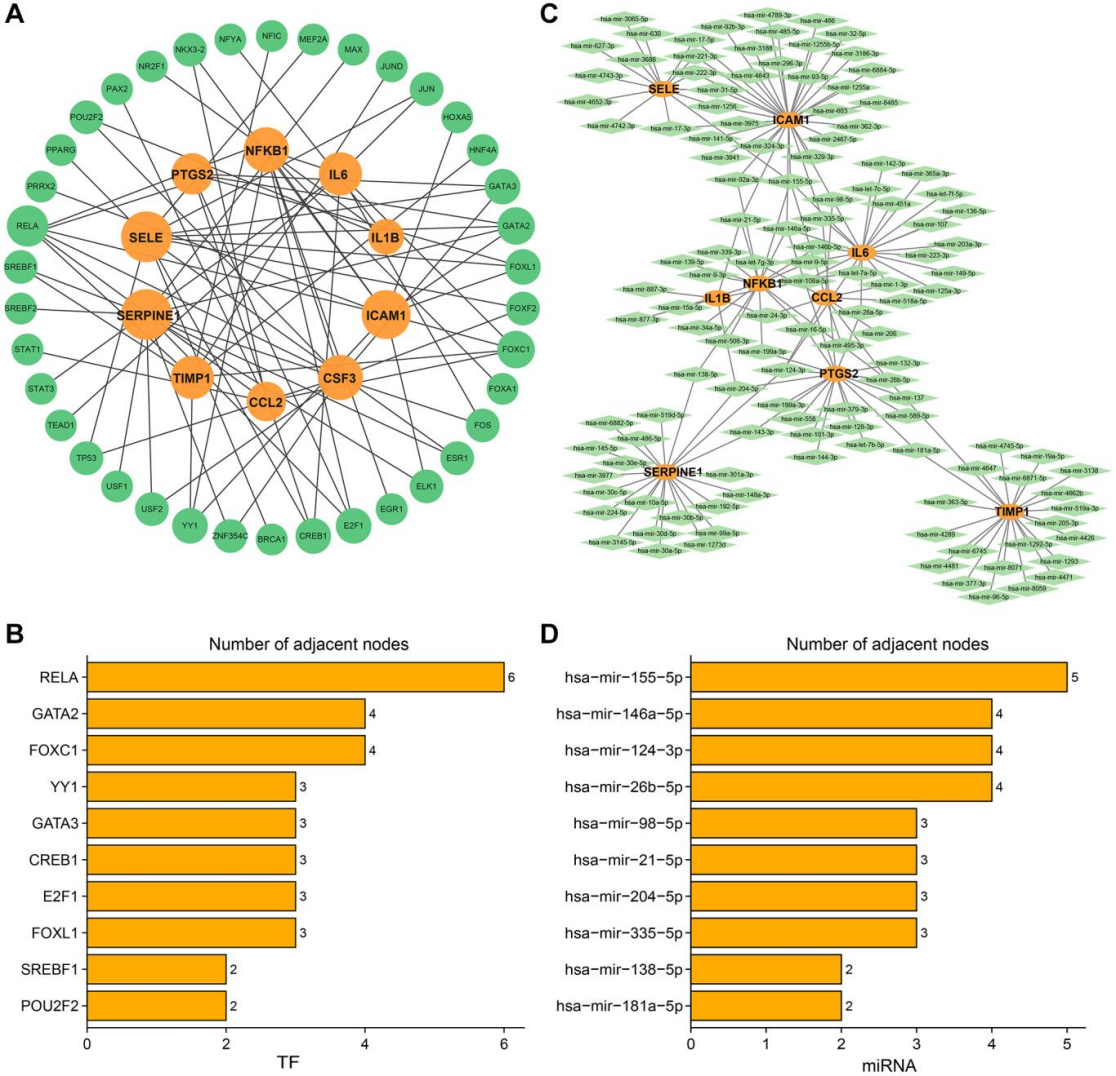
**Transcription factors-hub genes and miRNA-hub genes regulatory networks in subtype B.** (**A**) TF-hub genes network. The orange circles indicate hub genes and the green circles indicate TFs. (**B**) Ranking TFs based on degree centrality. (**C**) miRNA-mRNA regulatory network. The orange ellipses indicate hub genes and the green rhombuses indicate miRNAs. (**D**) Ranking miRNAs based on degree centrality. The number of adjacent nodes.

In subtype B, a total of 127 miRNAs and 38 TFs were predicted. The TF-hub gene interaction network is shown in the figure (Figure 11A). Orange nodes and green nodes indicate hub genes and TFs, respectively. The graph shows the number of TF node connections. The RELA, GATA2, and FOXC1 node connection numbers were 6, 4, and 4, respectively, which are connected to more hub genes and may play important regulatory roles (Figure 11B). The miRNA-hub gene interaction network is shown in Figure 11C. The oval and diamond nodes represent hub genes and miRNAs, respectively. Among them, CSF3 had no miRNA prediction. Hsa-miR-155-5p, hsa-miR-26b-5p, hsa-miR-124-3p, and hsa-miR-146a-5p node connections were 5, 4, 4, and 4, respectively (Figure 11D), which may play an important role in the AMD pathogenesis pathway.

**Figure 12:**
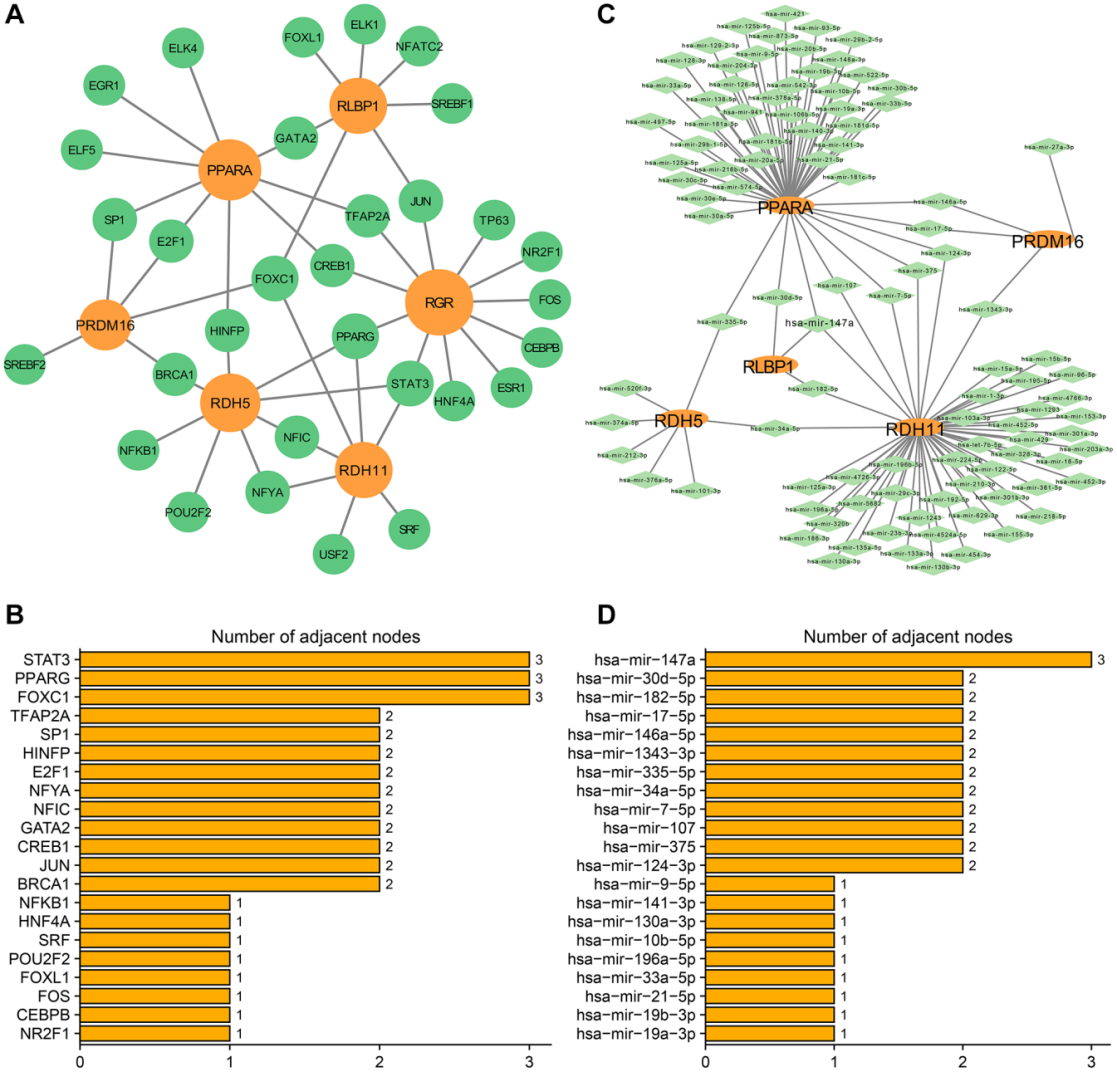
**Transcription factors-hub genes and miRNA-hub genes regulatory networks in subtype A.** (**A**) TF-hub genes network. The orange circles indicate hub genes and the green circles indicate TFs. (**B**) Ranking TFs based on degree centrality. (**C**) miRNA-mRNA regulatory network. The orange ellipses indicate hub genes and the green rhombuses indicate miRNAs. (**D**) Ranking miRNAs based on degree centrality. The number of adjacent nodes.

In subtype A, a total of 100 miRNAs and 31 TFs were predicted. The TF-hub gene interaction network is shown in the figure (Figure 12A). Orange nodes and green nodes indicate hub genes and TFs, respectively. Among them, RGR, PRDM1, RDH5, RLBP1, PPARA, and RDH11 hub genes interacted in the TF-hub genes network. The graph shows the number of TF node connections. The STAT3, PPARG, and FOXC1 node connection numbers were 3, which are connected to more hub genes and may play important regulatory roles (Figure 12B). The miRNA-hub gene interaction network is shown in Figure 12C. The oval and diamond nodes represent hub genes and miRNAs, respectively. Among them, PPARA, RDH11, RDH5, RLBP1, and PRDM16 hub genes interacted in the miRNA-hub genes network. Hsa-mir-147a node connections were 3 (Figure 12D), which may play an important role in the AMD pathogenesis pathway.

## DISCUSSION

In recent years, the role of cellular senescence in diseases has been extensively studied, and it has been found to play a significant role in several conditions, including AMD. Prior research has identified significant morphological changes in RPE cells during the aging process, where they increase in size and become multinucleated. If the enlarged cells fail to form multinucleation, they may undergo collapse [17].

This suggests that the development of AMD is associated with cellular senescence. We attempted to construct a diagnostic model for AMD using genes related to cellular senescence, as depicted in the random forest plot, aiming for a more timely and accurate identification of early-stage AMD. In the random forest analysis, we obtained five risk genes, namely, CCL8, VEGFA, CXCL10, IGFBP1, and AREG, based on their GINI importance scores. We utilized these genes to establish a diagnostic model. Furthermore, our predictive model demonstrated excellent predictive capabilities. CCL8 belongs to the CC chemokine family and serves as a chemotactic factor. It acts as a chemoattractant, recruiting monocytes, lymphocytes, eosinophils, and basophils, among others, thereby mediating inflammatory host responses [18]. CXCL10 is a ligand for CXCR3 and is produced in the ganglion cell layer (GCL) of the retina after ischemic injury. CXCL10, along with CXCR3, recruits microglia, monocytes, and activated T cells to the superficial retina. These inflammatory cells produce more cytokines and chemokines, further inducing neuronal death by activating apoptotic signaling pathways [19–21]. VEGFA is a growth factor that plays an active role in angiogenesis, vasculogenesis, and endothelial cell growth. It induces endothelial cell proliferation, promotes cell migration, inhibits apoptosis, and induces vascular permeability [22]. This is a critical factor in the development of neovascularization, which is a key reason for the formation of wet age-related macular degeneration (AMD) in its later stages. IGFBP1 is a member of the insulin-like growth factor binding protein family, and this protein plays a crucial role in cell migration and metabolism. It may also have immunomodulatory effects during the process of cellular senescence [23]. This suggests that IGFBP1 could serve as a new genetic marker for AMD. AREG encodes a protein that belongs to the epidermal growth factor family. AREG can be secreted by macrophages during chronic inflammation and plays a role in enhancing the immunosuppressive capacity of regulatory T cells [24]. The mechanism maintaining retinal immune privilege can be considered layered protection. When pathogens breach BRBs, the second layer of protection is triggered, inducing retinal cell tolerance to invading pathogens and suppressing immune cell activation [25]. This suggests that AREG may be involved in early retinal immune layered protection in AMD inflammation. VEGFA is associated with disease progression in AMD risk genes, while CCL8, CXCL10, IGFBP1, and AREG are related to the development of inflammation. These mediators further stimulate microglia or macrophages and the tissue complement system. Therefore, this diagnostic model may be constructed based on the inflammation induced by cellular senescence. Further research is needed to investigate the detailed mechanisms underlying these risk genes and their relationship with AMD.

Currently, existing treatment strategies for AMD involve anti-VEGF therapy. However, anti-VEGF strategies may not halt the progression of geographic atrophy, which can lead to vision loss over time. This suggests that targeting a single pathway alone may not be sufficient to prevent disease progression, and specific treatment approaches tailored to different subtypes are more likely to be effective [26]. We have been exploring alternative and more effective treatment approaches tailored to different subtypes of AMD at the transcriptome level. Group analysis of 27 senescence-related differential expression genes revealed that subtype B exhibited a higher degree of cellular senescence. Furthermore, based on GO, KEGG, and GSVA enrichment analyses, subtype B showed significant upregulation in terms of inflammatory responses, immune reactions, and programmed cell death, while it exhibited significant downregulation in components and biological functions related to maintaining normal cell functions, such as extracellular matrix, eye development, and epithelial cell differentiation. We conducted a more in-depth investigation into intergroup comparisons and found that the levels of cellular senescence and gene expression related to programmed cell death, including necroptosis, ferroptosis, and pyroptosis, were significantly higher in subtype B than in subtype A. This may further explain the pathogenesis of subtype B AMD, and treatment approaches targeting cellular senescence and programmed cell death pathways could offer greater benefits to subtype B patients.

To further explore the pathogenic mechanisms of the A and B subtypes and the heterogeneity between them, we performed WGCNA. We selected coexpression gene modules that best differentiate the functional differences between the two subtypes. The brown module showed the highest correlation and exhibited a positive correlation within the B subtype. The turquoise module showed the highest correlation and exhibited a positive correlation within the A subtype. To focus on the core biological functions of the coexpression module, we conducted a PPI analysis to investigate interactions between proteins and further identified functional hub genes within the network. Our analysis revealed that these module hub genes are widely associated with inflammatory responses and immunity. This indicates that the B subtype exhibits higher levels of immune system activation and infiltration than the A subtype.

To further explore the regulatory network among the module genes and intervene in the inflammatory progression of the B subtype, which exhibits a higher level of immune activation, we conducted transcription factor and miRNA regulatory network analyses on the hub genes. In the transcription factor regulatory network analysis, we identified widespread regulation of hub gene expression by NF-κB, FOXC1, and GATA2. These findings suggest their potential involvement in the immune infiltration and progression of the B subtype and highlight their potential as target molecules for precision therapy in the B subtype. RELA (RELA Proto-Oncogene, NF-KB Subunit) is a protein-coding gene. Research has revealed that in animal models and human AMD, NF-κB and STAT-1 may form a complex that jointly regulates LCN-2 expression in the retina, thereby stimulating an inflammatory response. The AKT2/NF-κB/LCN-2 signaling axis represents a potential therapeutic target for AMD [27]. Recent advancements suggest that kinases controlling NF-κB activation, such as the IKK complex, possess dual independent functions, as they also regulate cell death checkpoints [28]. Therefore, NF-κB may emerge as an effective therapeutic target for B-subtype AMD. FOXC1 belongs to the forkhead family of transcription factors and is characterized by a unique DNA-binding forkhead domain. This gene has been shown to participate in the regulation of embryonic and ocular development. Mutations in this particular gene give rise to a range of glaucoma manifestations, including primary congenital glaucoma, autosomal dominant iridogoniodysgenesis anomaly, and Axenfeld-Rieger anomaly. Currently, there is limited research on the transcriptional regulatory role of FOXC1 in AMD, but a study [29] has indicated its significant role in corneal epithelial development. Lack of FOXC1 may result in disruption of the corneal epithelium. Research on the function of this gene has primarily focused on its antiangiogenic effects in the corneal stroma [30–32]. Further investigation is required to elucidate the mechanisms by which FOXC1 functions in AMD. GATA2 is an activator of VEGFR2 transcription [33], and knocking down GATA2 using siRNA reduces the activity of the VEGFR2 promoter. This suggests that transcriptional regulation by GATA2 may be a contributing factor to the elevated expression of VEGFA, which in turn induces neovascularization in wet AMD. Controlling the regulation of GATA2 may offer a potential avenue for the treatment of wet AMD.

TFs (transcription factors) play a role in promoting or inhibiting transcription at the pretranscriptional stage [34], while miRNAs exert important regulatory functions at the posttranscriptional level [35]. Specific miRNA genetic markers may have utility in predicting the prognosis of AMD [36]. In the constructed hub gene-miRNA network, hsa-mir-155-5p, hsa-mir-26b-5p, and hsa-mir-124-3p were the miRNAs with the highest number of connections to hub genes. HSA-miR-155-5P is a miRNA that is overexpressed in retinal specimens of patients with advanced age-related macular degeneration (AAMD). Research by Jorgensen et al. [37] suggests that the progression of AAMD may result from immune and inflammatory response dysregulation associated with RPE damage, which is related to changes in MHC (major histocompatibility complex) and the complement system. The potential regulatory role of HSA-miR-155-5P miRNA in the immune response of AAMD is implicated through its interaction with MHC II targets. This evidence suggests an increased immune reactivity of HLA class II in retinal specimens of AMD patients, and HLA class II antigens are components of drusen [38]. This implies that HSA-miR-155-5P may play a potential role in the occurrence and development of AMD and could be further explored as a therapeutic target. Previous studies have suggested that HSA-miR-146A-5P may serve as a genetic marker for AAMD. In specimens from individuals without eye disease, the expression of this miRNA in the choroidal RPE is nearly 100 times higher than its expression in the neural retina [39]. Overexpression of HSA-miR-146A-5P in retinal specimens of AAMD is 2.1 to 6.3 times higher than that in age-matched control specimens without AAMD [40]. HSA-miR-146A-5P has a conserved, high-affinity, polymorphic seed pairing site in the 3′UTR of CFH, making it the strongest and most consistent among AAMD-associated genes [36]. Therefore, HSA-miR-146A-5P may play an important role in the pathogenesis of AMD and could be a significant target for future treatments. According to previous research, miR-26 has been identified as a novel factor in regulating the survival of rod photoreceptors and is involved in processes related to cell proliferation and apoptosis [41]. However, mechanistic evidence of miR-26b-5p in AMD is still limited. Mendes-Silva et al. reviewed miRNAs implicated in shared biological pathways between Alzheimer’s disease and depression [42]. From this review, it is known that miR-26b is involved in various signaling pathways, including the transforming growth factor beta receptor signaling pathway, T-cell receptor signaling pathway, epidermal growth factor receptor signaling pathway, intracellular protein transport, nerve growth factor receptor signaling pathway, neurotrophin signaling pathway, and cellular component disassembly involved in the apoptotic process. These pathways may be associated with immune mediation, inflammation, and cell apoptosis in the pathogenesis of AMD. Further research on miR-26b holds the potential to uncover the mechanisms underlying AMD. The mechanistic evidence for miR-124 in AMD is still limited. Given that both AMD and Alzheimer’s disease (AD) are neurodegenerative diseases and that amyloid-beta (Aβ) aggregates can be found in the retinas of AMD patients, it is possible to determine potential pathways for AMD through the study of AD [43]. Previous research has reported abnormal expression of miR-124 in the brains of AD patients [44, 45] and suggested that it may increase Aβ production by regulating the expression of BACE1 and/or amyloid precursor protein (APP) [46]. It has been confirmed that Aβ can upregulate the expression of miR-124 in the brains of TG2576 mice. The high expression of miR-124 in AMD may be related to the presence of Aβ deposits in drusen bodies in AMD patients [47]. Additionally, *in vitro* and *in vivo* experiments conducted by Marisetty AL et al. suggest that the miR-124 pathway regulates cell apoptosis [48]. This may imply that miR-124 is associated with apoptosis in RPE cells in AMD, making the miR-124 regulatory pathway a potential therapeutic target for B-subtype AMD. However, further research is needed to elucidate the specific mechanisms by which miR-124 functions in the context of AMD development.

In the A-subtype transcription factor regulatory network analysis, PPARG and STAT3 were associated with the transcription of RGR, RDH5, and RDH11 visual cycle-related genes. Previous studies have shown that the retinal pigment epithelium (RPE) interacts closely with photoreceptors to recycle retinal pigments and essential lipids and to exchange nutrients in the blood [49]. In AMD, the RPE is dysfunctional and the homeostatic processes of Bruch’s membrane degradation and rebuilding are disrupted. This results in the metabolic deposition that promotes the increase in Bruch’s membrane thickness [50]. Transcription of visual cycle genes such as RPE65, RGR, RDH5, and RDH11 has been shown to increase with age in the RPE. One of the age-related visual cycle genes, RDH5, has been identified as one of the 15 putative causative genes for advanced AMD; an SNP at the RDH5 locus (rs3138141) identified in the AMD GWAS affects the expression of this gene in the RPE [51]. The correlation between STAT3 and visual cycle-related genes such as RGR, RDH5, and RDH11 is still unknown, and we speculate that the deposition of lipofuscin with aging leads to retinal stress, which may be expected to be a new target for the treatment of subtype an AMD by activating the transcriptional regulation of visual cycle-related genes such as RGR, RDH5, and RDH11 by STAT3 transcription factors. Its specific mechanism needs to be further investigated.

The exact transcriptional mechanism of PPARG with RGR, RDH5, RDH11, and other visual cycle-related genes is still unknown. Peroxisome proliferator-activated receptor gamma (PPARgamma) is a member of the ligand-activated transcription factor nuclear receptor family, and after PPARs are activated by ligand binding, they form a heterodimer with retinal X receptor (RXR), The formed PPARγ/RXR heterodimer binds to the PPAR response element (PPRE) upstream of the promoter of the target gene and promotes or represses transcription of the target gene. The specific regulatory mechanism requires further investigation.

RLBP1 (retinaldehyde binding protein 1) is a protein-coding gene involved in the regeneration of active 11-cis-retinol and 11-cis-retinaldehyde, which are important components of the “visual cycle”. FOXC1, a gene belonging to the forkhead family of transcription factors, regulates the development of the nervous system by influencing the synthesis of retinoic acid by affecting the expression of the Rdh10 gene [52]. FOXC1 may be a key component of the retinaldehyde binding protein (RLBP). FOXC1 may be an important regulator of cell viability and resistance to oxidative stress in the eye [53]. Its exact regulatory relationship with RDH11 and RLBP1 remains unknown. PRDM16 transcriptionally regulates the differentiation function of brown adipose tissue (BAT), which consumes chemical energy exclusively as heat in response to cold or overfeeding. Drusen has been shown to contain acute phase proteins, C-reactive proteins, complement components, complement inhibitors, apolipoproteins, lipids, and many other proteins [54]. There is still no well-established study to illustrate the relationship between FOXC1 and retinal visual cycle-related genes and PRDM16 in AMD. We speculate that during the development of AMD, RPE dysfunction and elevated levels of FOXC1 transcription factors may regulate the expression levels of PRDM16, RDH11, and RLBP1 in response to retinal oxidative stress.

In the analysis of the subtype A miRNA regulatory network, hsa-miR-147a showed a correlation with PPAPA, RLBP1, and RDH11, and its specific mechanism of action is still unknown in AMD. Hsa-miR-147a may play a regulatory role in retinal inflammatory response, and oxidative stress process by inducing macrophage activation.

AMD has been studied by several institutions using the GSE29801 dataset through bioinformatics technology studies. By combining data from the GSE29801 and GSE135092 datasets, Dhanach et al. performed differential expression analysis and KEGG enrichment analysis, which revealed the two most significant and relevant biological processes in macular RPE/choroid tissue samples in AMD, namely the neuroactive ligand-receptor interaction pathway and the extracellular matrix-receptor interaction pathway. In addition, the protein-protein interaction (PPI) network identified two key genes involved in the control of cell proliferation/differentiation processes, HDAC1 and CDK1. Overall, this analysis provides new insights to expand the investigation of AMD pathogenesis by increasing the number of molecular determinants and functional pathways supporting AMD-associated RPE/choroid dysfunction [55]. Daoxin et al. selected RPE-choroid tissue samples from the GSE29801 dataset differentially expressed genes from normal and AMD patients and performed GO and KEGG enrichment analysis. The key modules and modular genes with the strongest association with AMD were screened by weighted gene co-expression network analysis (WGCNA). Based on the modular genes, the SVM machine-learning disease prediction model was finally constructed, and the disease signature genes constructed for the model were associated with abnormal glucose metabolism and immune cell infiltration. It may become a promising new target for targeted therapy of AMD [56]. Yu et al. identified differentially expressed mRNAs from GSE50195 and GSE29801, respectively, and based on literature review, Starbase database analysis, and RNA hybridization analysis, the authors obtained miRNA-mRNA pairs and circRNA-miRNA pairs. By combining these pairs, the authors constructed circRNA-miRNA networks. Using protein-protein network analysis, the MCODE algorithm, and the highest degree of circRNA nodes, the regulatory axis of hsa_circRNA7329/hsa-miR-9/SCD was identified, which may regulate SCD via hsa-miR-9 to promote macrophage-mediated inflammation and pathological angiogenesis, leading to the development of AMD. However, potential details require further investigation [57]. Zhiyue et al. analyzed the differentially expressed genes in AMD using GSE125564 and GSE29801 datasets. The upregulated differentially expressed genes were found to be mainly enriched in biological processes such as DNA replication, nucleoplasm, extracellular extracellular bodies, and calcineurin binding. In addition, dry AMD DRGs were mainly enriched in membrane components and blood-aqueous barrier (BAB) formation, which may shed light on the pathogenesis [58]. The uniqueness of our article is that we synthesized the RPE-choroid sample genes in the GSE29801 gene set with the senescence gene set “Senmayo”, and the clinical prediction model constructed at the cellular senescence level with the investigation of the pathogenesis of AMD provides potential new therapeutic targets for the treatment of AMD.

Although our study had a comprehensive analytical process, it still has some limitations. First, in our study, we searched for several therapeutic targets against subtype B AMD, we found RELA at the transcription factor level, and we found hsa-mir-155-5p at the miRNA level, but we still need to consider robust functional experiments to validate its pathogenic mechanism and therapeutic possibilities as a therapeutic target, and our group will follow up the experimental studies to further explore the pathogenesis of AMD and therapeutic targets. Second, the dataset is based on a retrospective analysis of public databases, and prospective studies are needed to investigate the pathogenesis of AMD and evaluate the predicted efficacy of therapeutic targets against subtype B in the future.

In summary, we constructed a diagnostic model for AMD based on the transcriptomic profile of cellular senescence-related genes. We classified AMD patients into two subtypes, A and B, based on their levels of cellular senescence. These two subtypes exhibit distinct immune microenvironments and biological characteristics. Through the analysis of transcription factor and miRNA regulatory networks, we identified potential regulatory mechanisms that may contribute to the phenotypic features of the B subtype. Furthermore, targeting these specific targets based on their regulatory pathways offers the potential to mediate the high cellular senescence levels and PCD-related features influenced by them in AMD. This provides valuable insights into the pathogenesis of AMD and potential avenues for future treatments.

## Supplementary Materials

Supplementary Figures
