# Supplementary material for: Bioinformatics analysis for constructing a cellular senescence-related age-related macular degeneration diagnostic model and identifying relevant disease subtypes to guide treatment
Source: Aging (Albany NY). 2024 May 10;16(9):8044–69. doi: 10.18632/aging.205804 (PMC11131993; doi:10.18632/aging.205804)
Supplement: Supplementary Figures [file aging-16-205804-s001.pdf]

SUPPLEMENTARY FIGURES

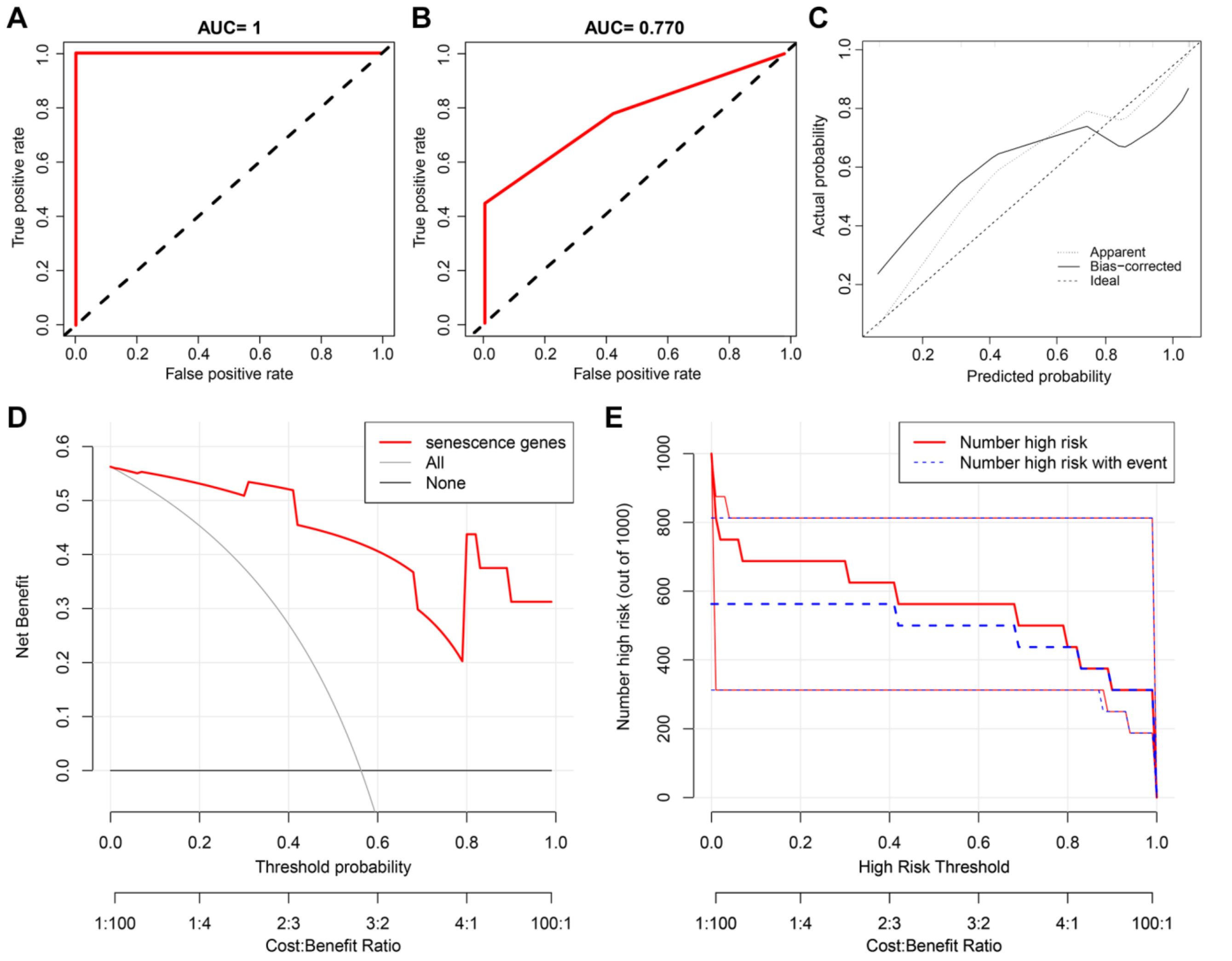

Supplementary Figure 1. The external validation of the diagnostic model. (**A**) The receiver operating characteristic curve (ROC) for the training set, AUC = 1. (**B**) The ROC for the validation set, AUC = 0.770. (**C**) Calibration curve of the validation set. (**D**) Decision curve analysis of the validation set. (**E**) Clinical impact curve of the validation set.

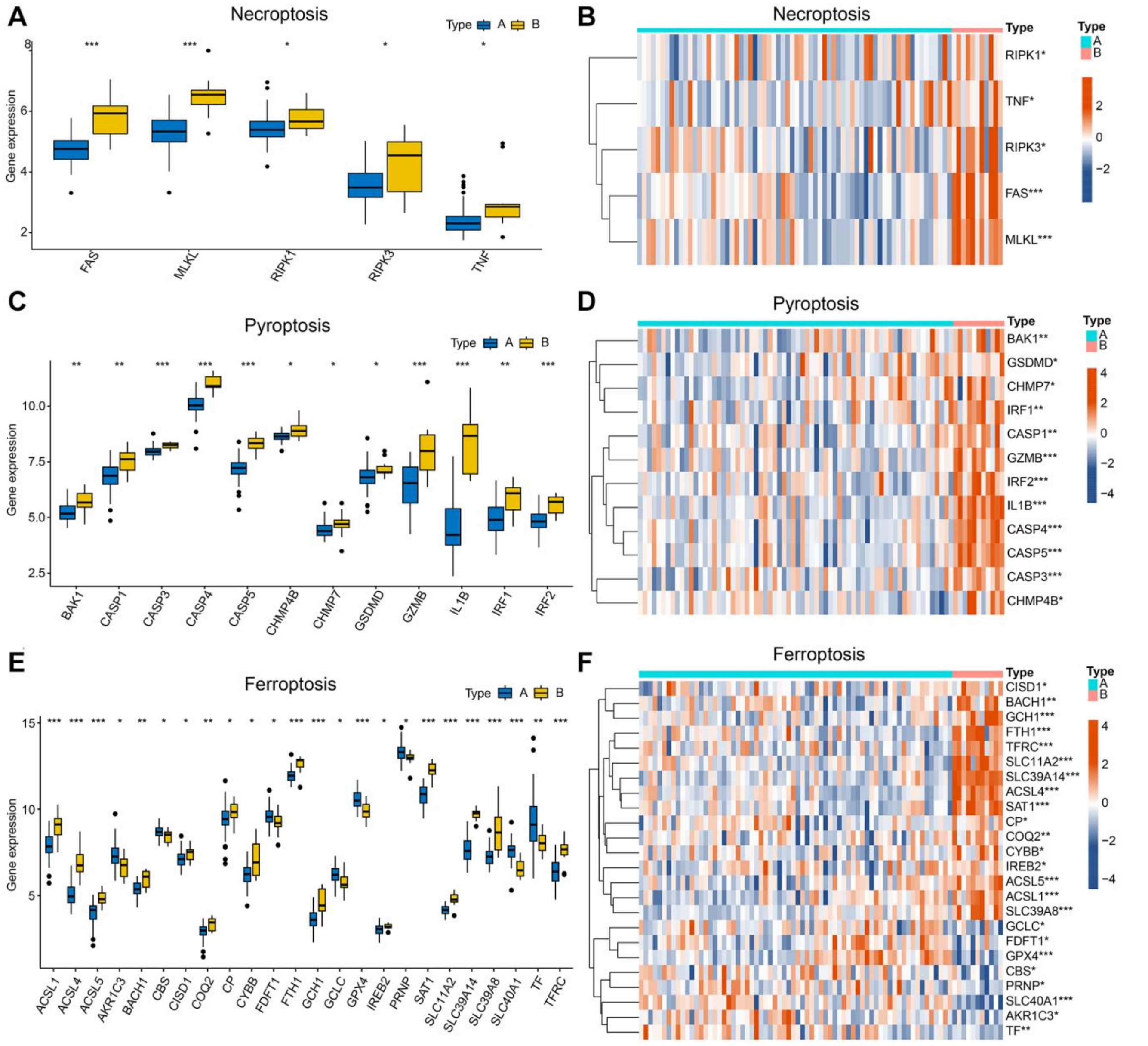

Supplementary Figure 2. Differential expression of subtypes A and B on multiple programmed death pathways. (**A**) Box plot of differential expression of A and B subtypes in Necroptosis. (**B**) Heatmap of differential expression of A and B subtypes in Necroptosis. (**C**) Box plot of differential expression of A and B subtypes in Pyroptosis. (**D**) Heatmap of differential expression of A and B subtypes in Pyroptosis. (**E**) Box plot of differential expression of A and B subtypes in Ferroptosis. (**F**) Heatmap of differential expression of A and B subtypes in Ferroptosis. ($^{*}P < 0.05$; $^{**}P < 0.01$; $^{***}P < 0.001$).

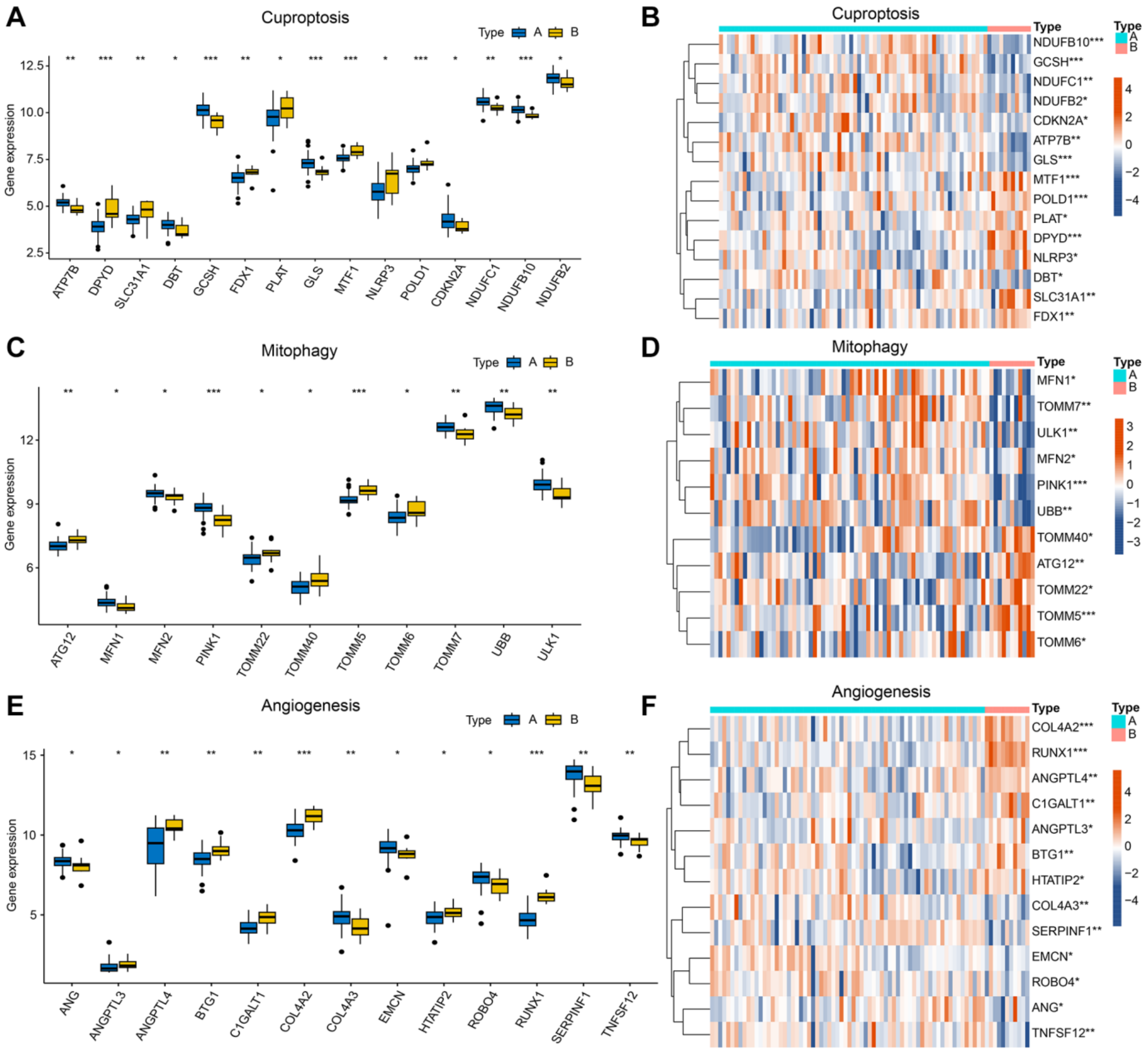

Supplementary Figure 3. Differential expression of subtypes A and B on multiple programmed death pathways and angiogenesis. (**A**) Box plot of differential expression of A and B subtypes in Cuproptosis. (**B**) Heatmap of differential expression of A and B subtypes in Cuproptosis. (**C**) Box plot of differential expression of A and B subtypes in Mitophagy. (**D**) Heatmap of differential expression of A and B subtypes in Mitophagy. (**E**) Box plot of differential expression of A and B subtypes in Angiogenesis. (**F**) Heatmap of differential expression of A and B subtypes in Angiogenesis. (*$P < 0.05$; **$P < 0.01$; ***$P < 0.001$).
